# Properties of Low-Cost WPCs Made from Alien Invasive Trees and rLDPE for Interior Use in Social Housing

**DOI:** 10.3390/polym13152436

**Published:** 2021-07-24

**Authors:** Abubakar Sadiq Mohammed, Martina Meincken

**Affiliations:** Department of Forest and Wood Science, University of Stellenbosch, Private Bag X1, Matieland, Stellenbosch 7602, South Africa; 23513187@sun.ac.za

**Keywords:** eco-friendly wood-based composites, wood polymer composites, bio composites, wall cladding, ceiling materials, RDP housing

## Abstract

Low-cost wood–plastic composites (WPCs) were developed from invasive trees and recycled low-density polyethylene. The aim was to produce affordable building materials for low-cost social housing in South Africa. Both raw materials are regarded as waste materials, and the subsequent product development adds value to the resources, while simultaneously reducing the waste stream. The production costs were minimised by utilising the entire biomass of *Acacia saligna* salvaged from clearing operations without any prior processing, and low-grade recycled low-density polyethylene to make WPCs without any additives. Different biomass/plastic ratios, particle sizes, and press settings were evaluated to determine the optimum processing parameters to obtain WPCs with adequate properties. The water absorption, dimensional stability, modulus of rupture, modulus of elasticity, tensile strength, and tensile moduli were improved at longer press times and higher temperatures for all blending ratios. This has been attributed to the crystallisation of the lignocellulose and thermally induced cross-linking in the polyethylene. An increased biomass ratio and particle size were positively correlated with water absorption and thickness swelling and inversely related with MOR, tensile strength, and density due to an incomplete encapsulation of the biomass by the plastic matrix. This study demonstrates the feasibility of utilising low-grade recycled polyethylene and the whole-tree biomass of *A. saligna*, without the need for pre-processing and the addition of expensive modifiers, to produce WPCs with properties that satisfy the minimum requirements for interior cladding or ceiling material.

## 1. Introduction

Alien invasive plants (AIP) in South Africa have enormous adverse consequences on biodiversity and ecosystem services and have led to direct environmental degradation [1,2]. With their high affinity for water and far-reaching roots and rapid spread, they often deplete water resources and degrade the soil, thereby water-stressing other plants and eventually alienating native flora and fauna in a local ecosystem. This has prompted the South African government to commit to a program aimed at clearing the most invasive plants. The cleared biomass is typically left behind to dry, where it poses a fire risk or, at best, is used as firewood. The issue of what to do with the biomass apart from the utilisation of fractions as firewood has emerged as a matter of concern.

Similarly, the surge in plastic pollution and its impact on the health and safety of ecosystems [3,4,5] is demanding attention. Plastic disposal in landfills results in leakages into the natural environment and consequently has dire adverse effects on wildlife, while incineration releases poisonous emissions. Plastic is therefore increasingly recycled, although the South African recycling industry is not quite as well developed as in Europe.

Making wood plastic composite (WPC) materials from these materials adds value to two different waste streams and can alleviate the financial burden of the government in managing these wastes and potentially form an employment- and income-generating enterprise by reprocessing them into secondary materials [5,6].

In South Africa, the government is trying to rectify the imbalance of property ownership caused by apartheid. Through the Reconstruction and Development Program (RDP), low-cost social housing units—called RDP houses—are being built across the country to address the enormous housing deficits [7,8]. However, the occupants of these budget houses are often exposed to the hazards of environmental stressors, such as heat and humidity, due to the low-quality of the building materials used [9]. The walls typically consist of a single-layer brick wall, without any additional insulation. Moisture condenses on the walls, resulting in mould, which increases their susceptibility to biodegradation and compromises the indoor air quality and health of occupants. The roofs are also often leaky, dripping water onto mostly wood or gypsum ceiling boards, which become soaking wet, develop discolouration, swell out of proportion, and deteriorate in a short time. These houses typically require maintenance shortly after construction, which the occupants cannot afford, resulting in undesirable living standards. Consequently, the use of interior ceiling and wall cladding materials that are high in hydrophobic properties to resist wetting-induced biodegradation while offering extended service lives is necessary. Interior cladding also offers the additional benefits of thermal and acoustic insulation against extreme weather conditions and community noise, which is typical in densely populated RDP settlements.

Utilising abundantly available recycled waste materials in the form of recycled low-density polyethylene (rLDPE) and AIPs as feedstock to develop low-cost WPC boards as wall cladding and ceiling material in RDP houses would add significantly to the living standard in these houses. The cost of the WPC materials is minimised by avoiding any unnecessary processing steps or additives and by using widely available raw materials that are otherwise regarded as waste materials that need to be removed. Recycled LDPE was chosen because of its abundant availability and low cost, as one of the main objectives was to keep the processing and manufacturing costs as low as possible. The recycled polymer was nominally linear low-density polyethylene (LLDPE), which has approximately the same density as LDPE, but with the linearity of HDPE and fairly short polymer chains. However, the obtained polymer was of the lowest quality. The plastic is sorted based on its polymer code and ranked into three quality grades—A, B, and C—in descending order. A-grade plastic is clean material generally sourced from shopping centres and recycled into pellets used for non-food packaging, such as refuse bags, furniture coverings, etc. C-grade is contaminated plastic that is often used in composite materials. Because of the impurities and the uncertainty of the exact composition, we refer to the recycled plastic material as rLDPE, rather than LLDPE.

After over a century since their first introduction, WPCs have evolved from wood-thermoset resin mixtures [10], through in-situ polymerization of plastic monomers within wood pores [10,11], to various blends of woody particles with thermoplastic polymers, as new thermoplastics with new properties and new technologies are continuously emerging [12]. The adhesion mechanism and bond strength between wood and plastic have been found to largely depend on the interlocking mechanism of a continuous plastic matrix through the wood cell lumen and intercellular pores, rather than the chemical bonds between polar wood cells and non-polar plastic [12,13,14]. Non-woody biomass, such as bark and leaves, are typically considered contaminants and avoided, because they generally lack the properties necessary for strength and bond formation [12,15,16]. Though leaves, bark, and twigs typically contain high amounts of lignin, which add stability, these are offset by the higher amounts of hemicelluloses, compared to woody biomass. Hemicelluloses are loosely bound non-crystalline fibres that oxidise easily and show a low thermal stability [12,13]. The inclusion of non-woody biomass into composites may thus lower their mechanical and thermal stability. In addition, leaves and bark are known to have a high extractive content [13,14,17]. Extractives are known to be rich in functional groups and affect the surface chemistry of wood [18]. Consequently, the bonds in WPC blends that depend on functional groups on the biomass surface may be enhanced by utilising biomass with a large extractive content. However, since polymers are largely unipolar, the majority of the bonds between wood and polymer rely on mechanical interlocking, rather than on chemical adhesion [12,19,20]. Contaminants may, however, add to the performance of WPCs. The higher lignin content in leaves and bark contributes to the moisture resistance, dimensional stability, and durability of composites [21,22] due to the hydrophobic nature of lignin and therefore provides some resistance against biological decay.

In recent reports, various kinds of non-wood lignocellulosic materials, including corncobs, nut shells [23], banana fibres [24], bamboo [25,26], rice husks [27], and many other unconventional biomass types, have found a use in WPC formulations. Owing to their chemical incompatibility, various protocols of pre-treatments have been applied in order to enhance interfacial bonding between biomass and plastic [13,14,28], which led to the introduction of coupling agents, such as maleic anhydride-grafted polypropylene (MAPP), Poly(ethylene-co-vinyl alcohol) (EVOH), and Polyethylene-graft maleic anhydride (PE-g-MA) to enhance bond strength [10,14,29]. However, coupling agents are costly and immensely increase the production costs of WPCs.

To manufacture WPC boards, temperatures between 140–200 °C [16,21,22,25,30] and press times between 5–30 min [17,23,24,31,32] are typically used. These press parameters significantly affect the properties of the WPCs. The upper and lower bound press temperature and time are usually given by the degradation temperature of the biomass type and the melting temperature of the thermoplastic [10,11,16,18]. Within these parameters are many possible combinations of processing settings, which affect the product properties.

Reports on the effect of biomass particle sizes and geometry on the properties of the resulting WPC boards are conflicting. Whereas some researchers report an improvement in some properties and a decrease in other properties with decreasing particle sizes [12,18,33,34,35], others conclude that particle size is less important to the final board properties than particle geometry [12,25,34,36,37]. Particle size reportedly affects the viscosity and melt flow rate during board formation [30,37,38] and consequently determines board–moisture relations.

Blending ratios between polymer matrix and wood reinforcements or fillers have been widely investigated [21,34,37,38,39,40,41] Depending on the intended properties and production method, ratios between 10% biomass, where the wood particles act as a filler in a continuous plastic matrix, and 90%, where the plastic acts as a binder, rather than a continuous matrix, have been reported. The biomass content at the higher extremes is usually the sole preserve of compression moulding methods, while the lower extremes may be injection moulded or extruded. A higher wood ratio generally results in an increased strength and stiffness, but it may also result in an increased moisture sorption and risks of microbial invasion [24,42]. 

In a previous study, the use of biomass obtained from *A. saligna*, *A. mearnsii* and *E. camaldulensis*—all invasive trees in South Africa—to produce WPCs was investigated [43]. The biomass was added in two forms: wood only and particles obtained by chipping and milling the entire tree with leaves, twigs, and bark. The results showed that regardless of the biomass type, WPCs made with *A. saligna* had superior mechanical properties, while composites with wood-only biomass were found to have better mechanical properties than composites with whole-tree biomass. In order to explain the superior properties of *A. saligna*, a chemical analysis was conducted on these species to identify the origin of the different properties of the resulting WPCs. Furthermore, it was essential to determine the optimum processing parameters to make WPC boards from entire trees of *A. saligna* and rLDPE. Consequently, different wood to plastic ratios and particle sizes were analysed, as well as different press temperatures and times. The physical and mechanical properties of the resulting WPC boards were analysed, and the effect of processing parameters on the performance of the final composites was determined.

## 2. Materials and Methods

### 2.1. Materials

**Figure 1:**
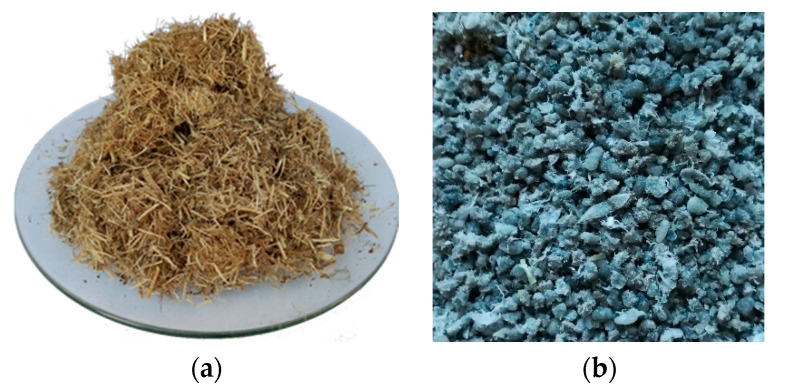
(**a**) Milled whole-tree biomass of *A. saligna* and (**b**) rLDPE.

*Acacia saligna* (Port Jackson) was obtained from clearing operations in the Western Cape of South Africa. Without any further processing, the trees were chipped, including wood, bark, leaves, seeds, and twigs, and air-dried (Figure 1a). The resulting biomass was passed through a 2 mm screen in a hammer mill and further air-dried to about 12% MC. Low-grade recycled low density polyethylene (rLDPE) consisting mostly of recycled shopping bags conglomerates, shown in Figure 1b, with a density of 0.915–0.950 g/cm^3^, was sourced from Atlantic Plastic Recycling (APR) CC, a plastic waste recycling plant in Cape Town, South Africa.

### 2.2. Composite Preparation

**Figure 2:**
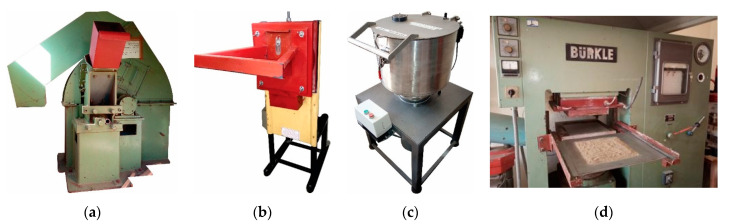
(**a**) Knife chipper, (**b**) hammermill, (**c**) custom-built blender, and (**d**) hydraulic press.

**Figure 3:**
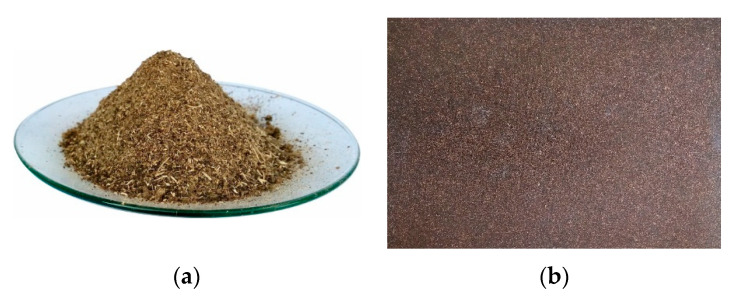
(**a**) Dry-compounded feedstock and (**b**) sample board, after hot-pressing at 180 °C for 30 min.

The biomass was chipped in an OC1 knife chipper (Figure 2a) from Heemaf, Netherlands and milled in a S1 hammermill from Drotsky, South Africa (Figure 2b) to a 2 mm particle size. The raw materials were mixed at three different biomass to plastic ratios—50:50, 60:40, and 70:30—and subsequently compounded in a custom built blender, shown in Figure 2c. The blending process resulted in a frictional breakdown of the WPC granules (Figure 3a), which were size separated into two fractions and characterised according to the procedure described in Section 2.3, from which the WPC boards were pressed.

The WPC granules were hot pressed at 150 and 180 °C for 10 and 30 min alternately in a hydraulic press from BURKLE, Germany in a 25 × 25 × 4 mm mould, shown in Figure 2d). The resulting boards shown in Figure 3b) were labelled with a three-digit sample code, where the first digit denotes the wood content (5, 6 and 7 = 50, 60 and 70 wt.%), the second letter identifies the particle size (*S* = 0.31 mm and *L* = 0.47), and the third letter (A, B, C, or D) identifies the time–temperature combination, where A: t = 10 min and T = 150 °C; B: t = 30 min and T = 150 °C; C: t = 10 min and T = 180 °C; and D: t = 30 min and T = 180 °C. Table 1 lists the processing parameters.

### 2.3. Size Analysis of Dry-Compounded Feedstock

**Figure 4:**
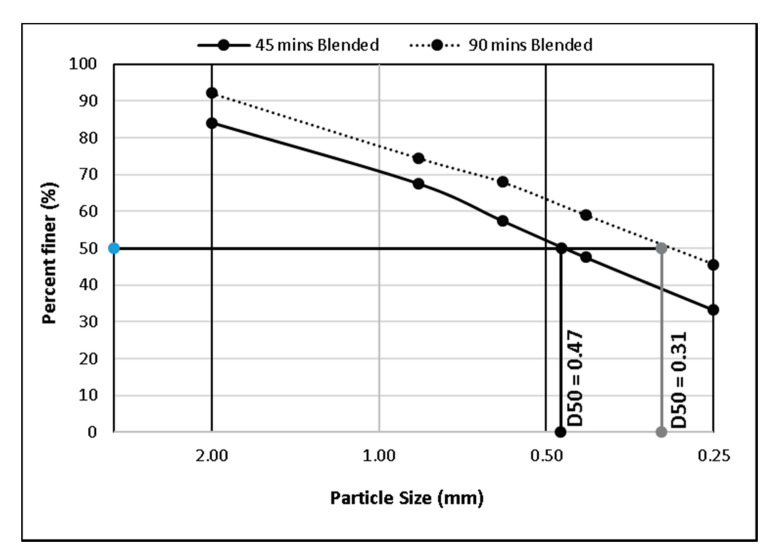
Particle size distribution, showing the variation in D50 as a function of the compounding time.

Blending of the rLDPE and biomass in a rotary drum compounder resulted in a further frictional fractionation into particle sizes, which are a function of the blending time. The raw materials were compounded for 45 and 90 min, resulting in two different particle fractions designated as ‘*L*’ for larger particles from the 45 min compounding time and ‘*S*’ for the smaller graded particles obtained from the 90 min blending cycle. For a better description of the particle size distribution, samples of 150 g from the compounded feedstock were characterised through sieve analysis in an AS 200 shaker from Retsch, Germany the details of which are presented in Figure 4, indicating D50 as the particle diameter below which 50% of the particles were located.

### 2.4. Physical Properties

The specimens were conditioned at 20 °C and a 65% ± 5% relative humidity for 4 weeks, prior to testing. Two boards were produced for each treatment, and eight samples per treatment were tested to obtain a mean and a standard deviation. The moisture content (*MC*) was determined in accordance with ASTM D4442-07, using Equation (1):

(1) $$MC\;(\%)=\frac{Original\;mass\left(g\right)-Ovendry\;mass\left(g\right)}{Ovendry\;mass\left(g\right)\times 100\;}$$

The water absorption (*WA*) and thickness swell (*TS*) of 16 samples per treatment were carried out in accordance with ASTM D1037-12. The specimens were immersed in distilled water for 24 h, 7-, 14-, 21-, and 28-day immersion periods. At the end of each immersion period, the specimens were removed from the water and weighed to the nearest 0.01 g, and their thickness was measured with a digital calliper to the nearest 0.01 mm. The values for *WA* were calculated according to Equation (2):

(2) $${WA}_{t}\;(\%)=\frac{W_{t}-W_{0}}{W_{0}}\times 100$$

where *WA*_t_ is the water absorption (%) at time *t*. *W*_0_ and *W_t_* are the respective weights of the specimens prior to immersion and after a given immersion time *t*.

*TS* was determined according to Equation (3):

(3) $${TS}_{t}\;(\%)=\frac{D_{t}-D_{0}}{D_{0}}\times 100$$

where *TS*_t_ is the thickness swelling (%) at time *t*. *D*_0_ and *D*_t_ are the respective thicknesses of the specimens prior to immersion and after a given immersion time *t*.

The board densities were determined according to Equation (4), as prescribed by ASTM standard D2395 (2014):

(4) $$\mathrm{Density}\;(\rho )=\frac{Ovendry\;mass}{airdried\;volume}$$

### 2.5. Chemical Properties

**Figure 5:**
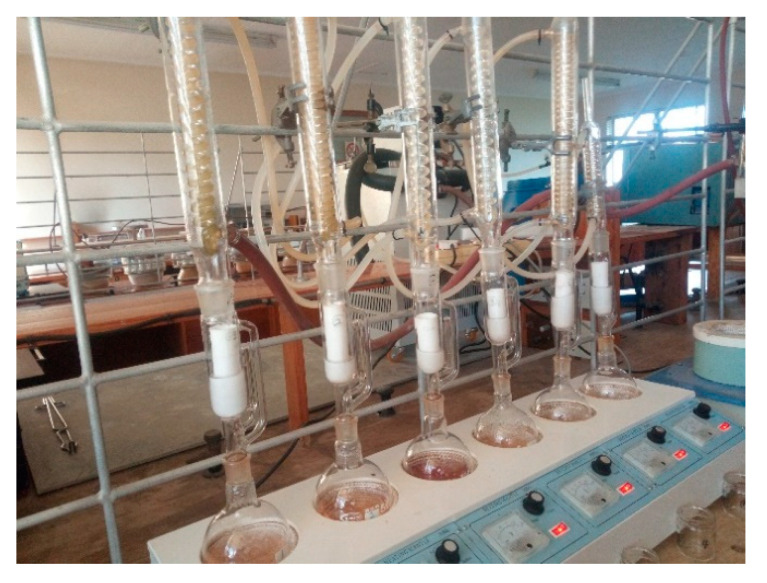
Soxhlet extraction setup for biomass compositional analysis.

The compositional analysis of the biomass from *Acacia saligna*, *Acacia mearnsii*, and *Eucalyptus camaldulensis* was conducted in accordance with the standards, NREL (LAP) TP-510-42620, TP-510-42622, TP-510-42621, TP-510-42618, and TP-510-42623 (2008) for ash content, hot water- and ethanol-soluble extractives (setup shown in Figure 5), lignin, sugar, and bulk density in order to determine how the composition affects the physical properties and performance of the WPCs.

### 2.6. Mechanical Properties

#### 2.6.1. Static Bending

**Figure 6:**
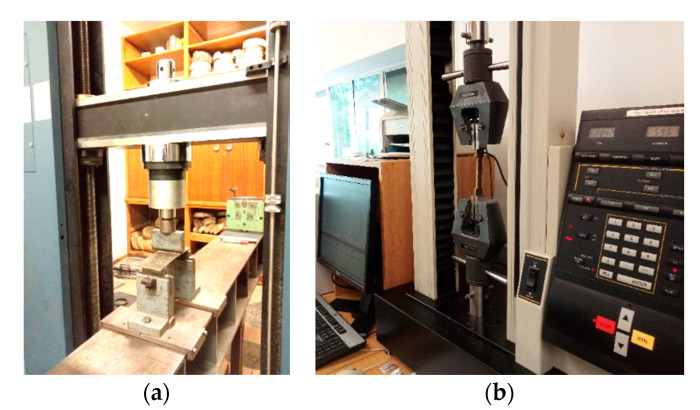
Instron setup for (**a**) the 3-point bending test, and (**b**) tensile test.

Four samples per treatment with a size of 195 × 50 × 4 mm were tested in a three-point bending test in accordance with ASTM D 1037-12 using a universal testing machine from Hottinger Baldwin Messtechnik (HBM), Germany (Figure 6a). The modulus of rupture (*MOR*) and modulus of elasticity (*MOE*) were determined according to Equations (5) and (6). All the calculated values are reported as the averages with the standard deviation.

(5) $$MOR=\frac{3P_{max}L}{2bd^{2}}$$

(6) $$MOR=\frac{L^{3}}{4bd^{3}}\frac{\Delta P}{\Delta y}$$

#### 2.6.2. Tensile Tests

Five dumbbell shaped samples with a size of 165 × 20 × 4 mm per treatment were tested in the tensile mode in accordance with ASTM D 638-14 using an Universal testing machine 4411 from Instron, Maine, USA equipped with a 5 kN load cell (Figure 6b), at a test speed of 50 mm/min. The tensile strength and tensile modulus were calculated based on Equations (7) and (8), and the average values are reported with the standard deviation.

(7) $$T_{S}=\frac{P_{max}}{bd}$$

(8) $$E_{t}=\frac{lg}{bd}\frac{\Delta P}{\Delta y}$$

### 2.7. Statistical Analysis

A factorial ANOVA with a replicates test in Statistica software 14.0.0.15 was used to determine significant differences within and between treatments at a significance level of α = 0.05. Pearson’s correlation analysis was conducted in R to quantify the contribution of all the independent variables on the physical properties of the boards.

## 3. Results

The findings from a previous study by Acheampong et al. [43], analysing WPCs made of biomass obtained from the whole tree or only the wood from *A. saligna*, *A. mearnsii* and *E. camaldulensis*, showed that the addition of bark, twigs, and leaves reduces the mechanical properties somewhat, but all boards still met the minimum requirements for interior use. A surprising finding, however, was that regardless of the biomass type, boards made with *A. saligna* showed significantly better mechanical properties than those made with the other two wood species. This was an unexpected result, especially the large difference between the two—otherwise very similar—Acacia species. To better understand the differences in performance, the chemical composition of the three different wood species was determined.

### 3.1. Chemical Composition

The chemical composition of the wood from *A. saligna, A. mearnsii*, and *E. camaldulensis* is presented in Table 2. *A. saligna* had the highest cellulose content of the three species, but with a low hemicelluloses content and average lignin content. The superior mechanical properties of WPCs made with *A. saligna* can be attributed to the high cellulose content, as the crystalline cellulose fibres are known to impart strength. The total extractive content of *A. saligna* was higher than that of the other two species and appeared to have no negative effect on the strength properties. On the contrary, the increased extractive content seemed to have aided the bonding between the biomass and the polymer matrix.

Based on the chemical composition and the results from Acheampong et al. [43], boards for further analysis were made from the entire tree of *A. saligna,* and the processing parameters were optimised to obtain the best physical and mechanical properties.

### 3.2. Water Absorption (WA) and Thickness Swelling (TS)

**Figure 7:**
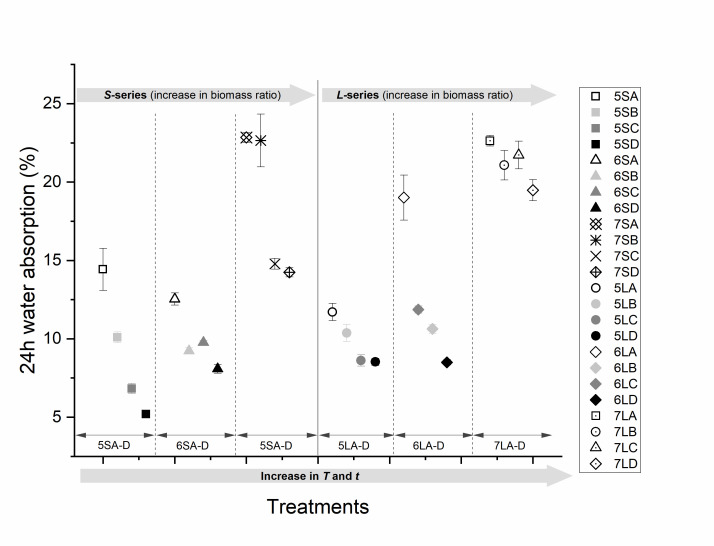
A 24 h WA of WPCs pressed at different temperatures and times (A–D) with 50, 60, and 70% biomass at two particle sizes (*S* and *L*).

Observations at 24 h WA of all WPCs (Figure 7) indicate that regardless of the compounding ratio and particle size, the water absorption of all treatment ‘*A*’ samples pressed at *T* = 150 °C and *t* = 10 min was the highest, while the WA of treatment ‘*D*’ samples pressed at *T* = 180 °C and *t* = 30 min was the lowest. This is because at 180 °C, the biomass acquires a hydrophobic character due to thermal modification, which leads to the bonding of amorphous hydroxyl groups and a reduction of the available bonding sites for water [44,45]. The higher temperature also leads to a reduction in the melt–flow viscosity of rLDPE [14,46]. With a melt temperature of maximum 140 °C, the melt flow rates of the rLDPE at 180 °C would have been high. An increased flow rate and extended pressing time will increase the potential of the polymer to infiltrate the cell wall micro pores, impregnate the cell lumen and encrust entire particles, therefore decreasing the water absorption. At 150 °C, however, which is barely above the melting temperature of the rLDPE, the plastic remained more viscous and left more hydroxyl groups on the wood fibre surface exposed as potential binding sites for water [16,46,47]. While an increase in either the press time (treatment B) or temperature (treatment C) resulted in a reduced *WA*, there was no clear trend as to which of the two factors has more impact in reducing *WA*. However, increasing both factors resulted in the lowest *WA*.

Figure 7 shows the *WA* for small (*S*-series) and large particles (*L*-series) at increasing biomass ratios. The trend indicates that an increase in the biomass ratio from 50–70% resulted in a linear increase in *WA* from below 15% to over 20%. As the biomass is hydrophilic and the plastic is hydrophobic, an increase in the biomass to plastic ratio means more available potential binding sites for water, which will increase the *WA*.

**Figure 8:**
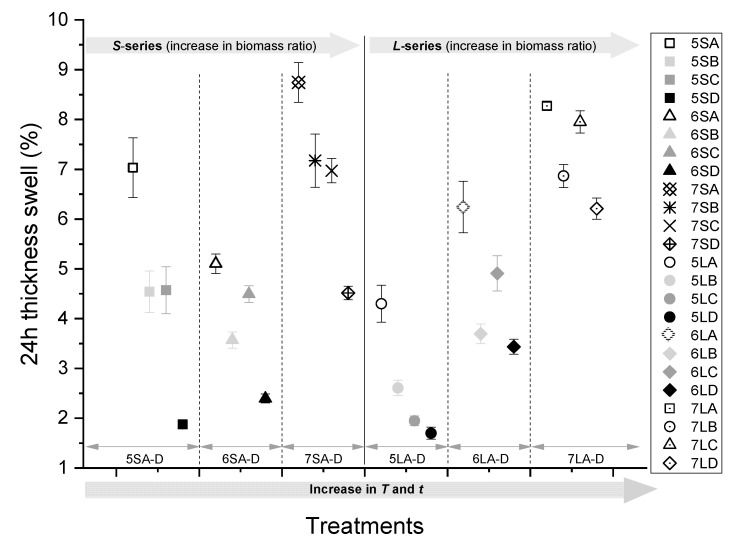
A 24 h TS of WPCs pressed at different temperatures and times (A–D) with 50, 60, and 70% biomass at two particle sizes (*S* and *L*).

Similar to the observed trends in WA, the TS (Figure 8) was independent of the particle size, and the longest pressing time and highest temperature resulted in the lowest TS across all blending ratios. While the increase in the press temperature or time alone resulted in an increased dimensional stability, none of the two factors showed any superiority over the other; however, the effect of the combined increase in press temperature and time resulted in the lowest TS (below 2%).

The short-term (2 h) and long-term (672 h) *WA* and *TS* for the various press settings (A–D) are presented in Table 2 for all blending ratios and particles sizes. As can be seen, the moisture absorption for press temperatures of *T* = 150 °C and *t* = 10 min (A) was the largest, particularly for WPC samples with a 70% biomass, which exceeded 30% MC after water immersion for 672 h. At press settings of *T* = 180 °C and *t* = 30 min (D), the lowest WA was recorded. WPCs made with larger particles generally absorbed more water than those made with smaller particles.

Board densities (Table 3) are a direct function of the blending ratio affected by the press temperature and time combination. Samples with a 50% biomass were consistently denser for all press settings (A–D) than WPCs made with 60% or 70% biomass, which had the lowest density. The presence of non-woody biomass in the form of leaves, twigs, and bark significantly reduced the density of the entire biomass below that of the rLDPE. The leaves with their characteristically thin cell walls, numerous intercellular spaces, and loosely packed spongy cells may not have contributed much to the weight but contributed enormously to the bulk volume of the biomass. Consequently, an increase in the biomass ratio resulted in an increase in the filler volume, without a proportionate increase in the mass. The combined effect of a high temperature at 180 °C and longer press time at 30 min under a 250 kg/cm^2^ pressure created a suitable condition for a reduced shear viscosity and improved the melt flow rate of the rLDPE, which generally has a melting temperature of 140 °C. The resulting boards were thus more compressed due to the better plastic melt-flow kinetics, and the smaller sized biomass particles allowed for a better compaction and improved the densification [15,35]. At a 70% biomass content, however, the plastic played the role of a binder, rather than a matrix. Since the biomass has a lower density than the plastic, a plastic weight ratio of 30% resulted in a much lower volume ratio, compared to the biomass. This disproportionate volume ratio implied that longer pressing times and higher press temperature were required for the plastic to melt and wet the biomass fibre surfaces, before successfully binding the particles together.

**Figure 9:**
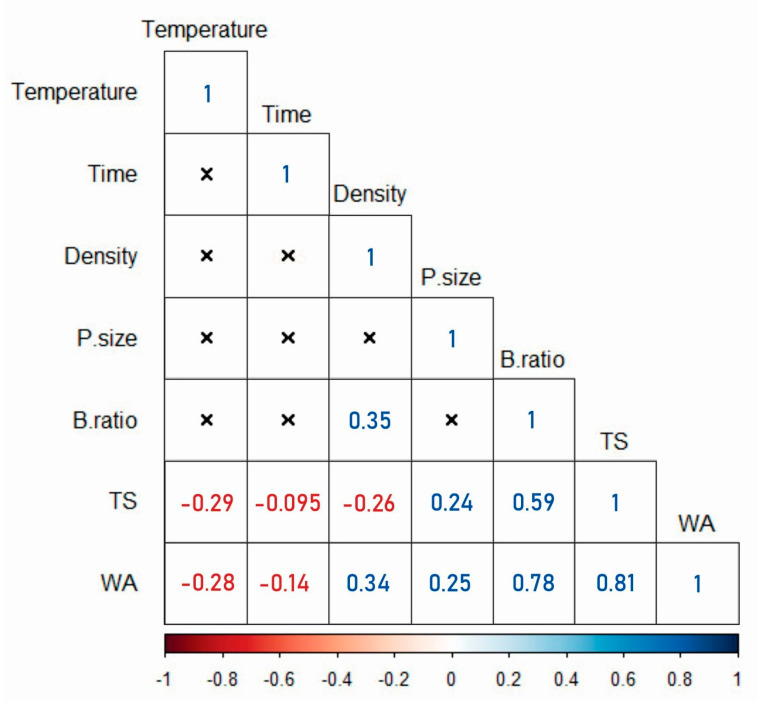
Pearson’s R correlations of the impact of independent press parameters on the physical properties (WA, TS and density) of WPCs. The correlation matrices marked (x) are not significant or inapplicable.

From the Pearson’s correlation matrices shown in Figure 9, the blending ratio (*B. ratio*) has the highest correlation (0.78) for WA among all the independent input variables and is therefore the most important determinant of water absorption. Since TS is a direct function of WA, as seen from Figure 9, the dimensional stability of the boards is also largely impacted by the blending ratio. As the press time and temperature are increased, WA and TS are decreased. Figure 7 and Figure 8 do not show clearly which of these two variables has a more significant impact on WA and TS. However, in Figure 9, it can be seen that temperature has a larger effect on the reduction of WA and TS than time. While press time is negatively correlated with TS, the correlation is not statistically significant. The effect of particle size on both WA and TS is also significant and positively correlated. Press temperature and time bear no significant correlation with density on their own, but the combined effect is significant. However, the blending ratio correlates positively with density. The board density shows a linear relation with WA and an inverse relation with TS.

### 3.3. Mechanical Properties

#### 3.3.1. Static Bending Strength

**Figure 10:**
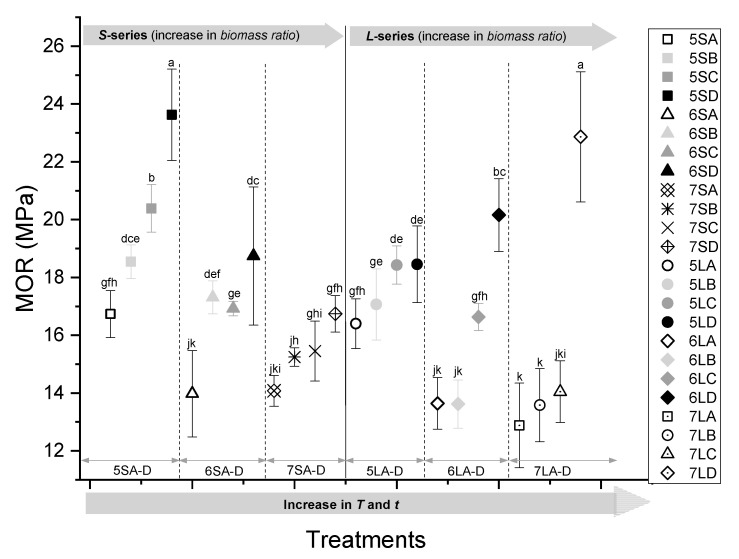
Flexural strength (MOR) of the WPCs pressed at different temperatures and times (A–D) with 50, 60, and 70% biomass at two particle sizes (S and L). Symbols with the same letters are not significantly different.

The results of the strength modulus of 3-point bending tests for all treatments are presented in Figure 10. For each blending ratio across particle sizes, the highest MOR is obtained for treatment ‘D’ samples pressed at *T* =180 °C and *t* = 30 min, while the lowest strength values were obtained for treatment ‘A’ samples pressed at *T* = 150 °C and *t* = 10 min. The effect of increasing the time (B) or temperature (C) alone resulted in an increase in strength, but there was no clear trend in terms of which of the two variables has a greater impact on strength. However, the increased press time and temperature together resulted in the highest board strength. This can be explained by the formation of a continuous polymer matrix around the biomass fibres and occupation of intercellular voids. The densified composite will have fewer micro pores and fewer stress concentration points and therefore an increased strength.

Boards with a 50% biomass had the highest bending strength properties, with a maximum MOR of about 23 MPa. As the biomass content increased, the MOR was reduced and showed the lowest value of 13 MPa at 70%. This can be explained by the inhomogeneity of the biomass, which contained fibres from leaves, twigs, and bark, which have a lower content of crystalline cellulose and higher portions of short fibres containing amorphous hemicelluloses and lignin. On the whole, boards made with a smaller particle size biomass (*S*) show slightly higher strengths than larger particles (*L*), although the difference is not statistically significant. This can be explained by the better flow of the polymer around smaller particles, which leads to a continuous matrix.

#### 3.3.2. Flexural MOE

**Figure 11:**
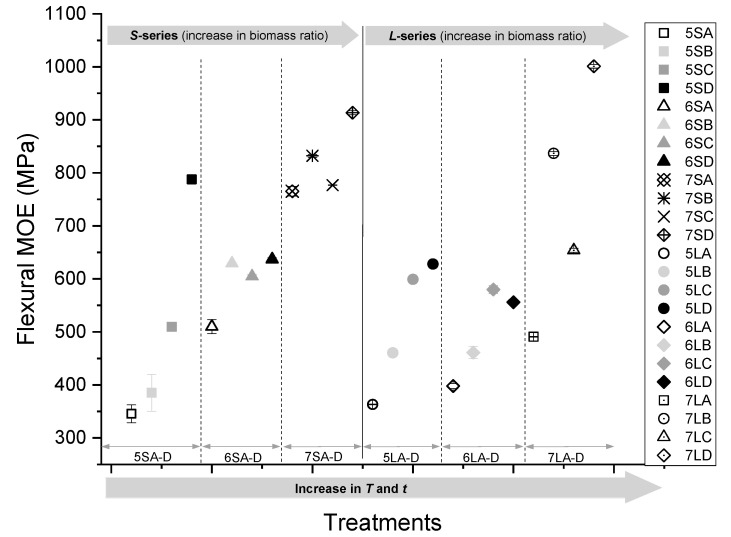
Flexural MOE of the WPCs pressed at different temperatures and times (A–D) with 50, 60, and 70% biomass at two particle sizes (*S* and *L*).

Figure 11 illustrates the bending stiffness of the WPC boards. Similar to the MOR, samples ‘D’ pressed at *T* = 180 °C and *t* = 30 min showed a superior bending stiffness for all blending ratios and particle sizes, while treatment samples ‘A’ pressed at *T* = 150 °C and *t* = 10 min recorded the lowest stiffness. As observed before, an increase in either the temperature or time of pressing resulted in an increase in stiffness, but without a clear trend regarding which of the two variables had a greater impact. However, unlike the MOR, composites with a higher biomass content at 70% show the highest bending stiffness, exceeding 900 MPa, and boards with a 50% biomass recorded the lowest stiffness. This indicates that the main contributor to stiffness is the biomass, which acts as a reinforcement in the composite. The impact of particle size on bending stiffness did not follow a clear trend, even though comparative observations of treatment ‘A’ and ‘D’ samples across particle sizes indicate that smaller particle sizes generally had better bending moduli.

#### 3.3.3. Tensile Strength

**Figure 12:**
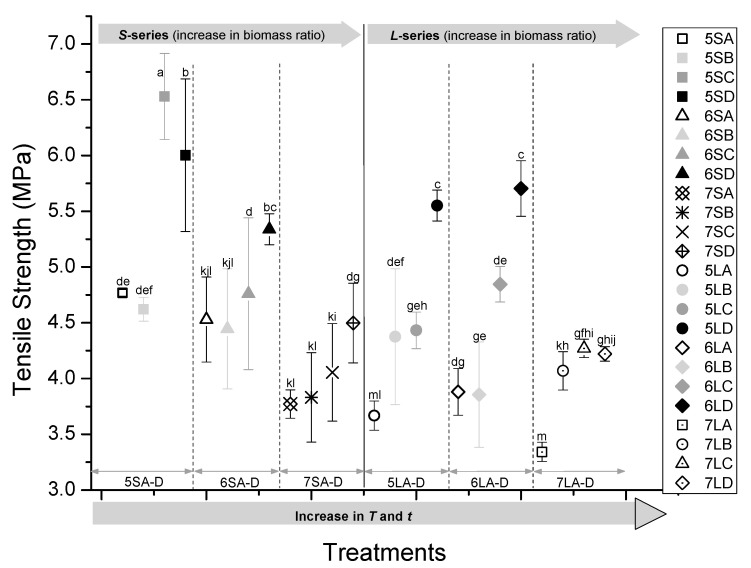
Tensile strength of the WPCs pressed at different temperatures and times (A–D) with 50, 60, and 70% biomass at two particle sizes (*S* and *L*). Symbols with the same letters are not significantly different.

The tensile strength of the WPC boards is illustrated in Figure 12 and shows a positive linear relationship with increasing press temperature and/or time. This trend can be explained by the more efficient matrix formation of the rLDPE around the biomass particles. The sustained elevated temperatures above the melt temperature of the rLDPE rendered it less viscous and improved its flow and diffusion into the cell wall micro pores to form continuous matrices, thus imparting a higher tensile strength. An inverse relationship between the tensile strength and biomass ratio was observed across particle sizes. At a higher biomass loading, the composite samples had a higher stiffness due to the reinforcing impact of the biomass. Consequently, an increase in the tensile load broke the samples with a higher biomass ratio at smaller strain levels. With a reduction in the biomass content, the WPCs gained elasticity, which resulted in a higher tensile strength. This phenomenon was observed across particle sizes. However, no clear trend regarding the independent effect of particle size on tensile strength could be statistically established.

#### 3.3.4. Tensile MOE

**Figure 13:**
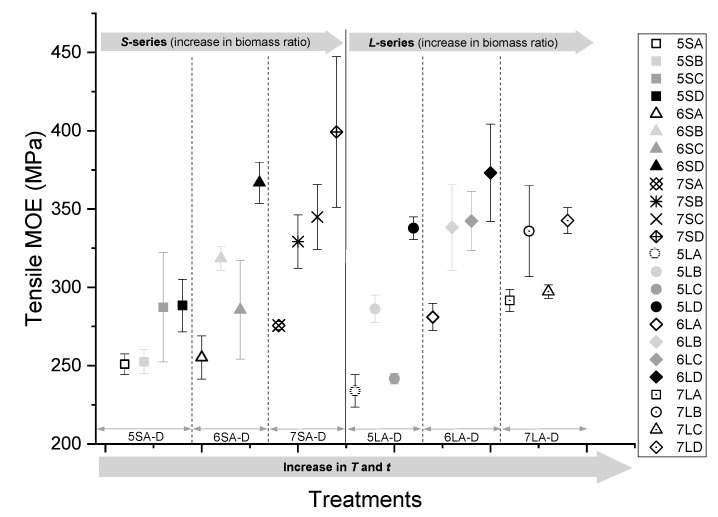
Tensile MOE of the WPCs pressed at different temperatures and times (A-D) with 50, 60, and 70% biomass at two particle sizes (S and L).

The observed tensile moduli were not very different from the tensile strength. From Figure 13, it is observed that treatment ‘D’ samples pressed at *T* = 180 °C and *t* = 30 min had a significantly higher tensile modulus than treatment ‘A’ samples pressed at *T* = 150 °C and *t* = 10 min for all blending ratios. This may be attributed to the re-crystallisation of cellulose, in addition to the realignment of lignin at a higher temperature and extended pressing time. However, an increase in the biomass ratio resulted in a rise in tensile stiffness, with the lowest stiffness recorded at approximately 230 MPa for a biomass ratio of 50% wt. and the highest stiffness of nearly 400 MPa at a 70% biomass ratio. The tensile modulus of the WPC is therefore largely determined by the blending ratio and press time and temperature. The effect of particle size generally showed no clear trend. However, the comparative analysis of treatment ‘A’ and ‘D’ samples across particle sizes suggests that the WPCs made with larger particles had higher tensile moduli.

## 4. Discussion

Given that cellulose plays an important role in bond formation [17,37,48], the higher content of cellulose in *A. saligna* suggests its higher potential for wood-polymer bond formation. However, this also implies a more hygroscopic character of the composite. This correlation between cellulose content and a high moisture uptake is confirmed by several researchers [12,15,18,31,47,49,50]. In order to maximise the full benefits of a high cellulose content, while simultaneously limiting the disadvantages, the press conditions that promote an efficient biomass encapsulation by the hydrophobic plastic are required. This is achieved at temperatures above the melting point of the plastic and a sufficient press time to allow for an effective flow, wetting, and diffusion into biomass. This can be observed in Figure 7, Figure 8, Figure 9, Figure 10, Figure 11, Figure 12 and Figure 13 and Table 3, which show that the elevated temperature and longer press time create better conditions to lower the viscosity of the plastic, therefore allowing it to reach into inter-particle crevices and cell lumina. The resulting formation of a continuous plastic matrix enhances the biomass particle encapsulation, which inhibits the bonding of water molecules to the free hydroxyl groups of holocellulose [15,47,50]. Subsequently, a reduction in water absorption and improvement in dimensional stability are achieved. This is further facilitated by the plasticisation of lignin and the conformational reorganisation of holocellulose due to the dehydration reactions of residual water resulting from the exposure of the biomass to high press temperature conditions [44,45,51]. These findings have been confirmed by several other researchers [14,52], who noted that the choice of press temperature and time affect the melt temperature and melt flow rates of the plastic and the thermal degradation temperature of the biomass [16,29]. The combination of the press temperature and blending ratio ultimately determines the extent of the particle encapsulation within the plastic matrix.

Extractives play a crucial role in wood–plastic bond formation. Polyphenolics and terpenes, including suberin and resin compounds, have hydrophobic functional groups with a considerable influence on the surface chemistry of wood [12,53,54,55]. Since a higher surface energy is required for a good bonding between biomass and polyethylene, a lower extractive content reduces the potential of wood–plastic adhesion via chemical bonds, while promoting mechanical interlocking. Some components of wood extractives oxidise and form volatiles upon exposure to air and light during drying and subsequent heat exposure during processing. This potentially lowers the surface energy of the biomass as extractives, particularly tannins, which are known to be present in considerable quantities in *A. saligna*, contribute largely to bond formation and water repellence. The reduced surface energy translates into a reduction in the adhesion potential [14,15,52]. At optimised press temperatures, however, extractives do not entirely escape, but migrate to the surfaces of the biomass, where they increase the adhesion between the plastic and biomass. As extractives migrate to the surface, they leave behind micro-voids, which, given sufficient press time will be filled by the plastic matrix through diffusion, which is a slow process [17,50,51,56]. The eventual wood–plastic bonds are both chemical at the interface and mechanical at the cell wall level. The high amount of extractives contained in the leaves and bark of the biomass also act as a plasticiser and can considerably reduce the viscosity of the plastic and enhance the particle dispersion in WPC processing [51,55,57,58]. These properties collectively enhance the moisture resistance of the composite products. Consequently, forming WPCs with biomass high in extractives requires the optimisation of the press settings to maximise both the benefits of the chemical adhesion via a surface energy boost, as well as mechanical interlocking.

At higher temperatures and longer press times, the hemicellulose concentration of the biomass, particularly of the leaves and twigs, may have either partially or wholly degraded. Boonstra et al. [59], Ates et al. [60] and Phuong et al. [61] have reported that the degradation of hemicelluloses starts below 200 °C. Inari et al. [62] found through X-ray photoelectron spectroscopy that after heat treatment at around 200 °C, the hydroxyl concentration of the wood surface decreased due to the thermal degradation of the hemicelluloses. Since the hydrophilic properties of wood depend on an amount of available hydroxyl groups, a reduction in the concentration of these functional groups results in an increased hydrophobic character of the wood [63]. These findings are corroborated by Hakkou et al. [45], Gérardin et al. [64], and Chaouch et al. [65], who reported that the oxygen to carbon ratio of wood at the surface decreases with increasing temperatures above 120 °C. This reduction in the O/C concentration ratio is accompanied by a reduction in the concentration of polar wood components due to dehydration reactions, the depolymerisation of amorphous holocellulose, and the selective degradation of hydroxyl groups. These reactions induce moisture resistance; however, they also lower the surface free energy of biomass and negatively affect wood polymer adhesion [17,59,66], while aiding apolar bonds [64].

Composites pressed at a higher temperature and time exhibited better mechanical properties. Thermal modification is known to induce crosslinks within wood and plastic, which explains the improvement of the mechanical properties. The increase in the stiffness of the WPCs with increasing temperature is in good agreement with the findings of other researchers, such as Lyutyy et al. [67] and Santos [68]. Kubojima et al. [69] noted that the MOE and MOR of wood increases for the first 30 min of heat treatment at 160 °C, after which it begins to decline. This has been attributed to lignin relocation and the re-crystallisation of cellulose [61]. However, the positive correlation between the increased press temperature and mechanical strength observed in this study was not confirmed by Hakkou et al. [45], Korkut et al. [70], or Bal and Bektaş [71], who reported that heating wood above 120 °C hindered the mechanical properties.

It was also observed that the dimensional stability and mechanical properties of the WPCs were better in samples made with smaller particles, irrespective of the compounding ratio. This can be explained by the more continuous matrix formation around the smaller particles. This observation is confirmed by various other studies [16,33,35,47,52], where the effect of the biomass particle size on the WPC properties was analysed. Smaller particles also result in better compressibility during hot pressing, which eliminates inter-particle voids and reduces the porosity of the composite. The enhanced compressibility also impacts directly on the board density, as better compaction results in a higher board density [30,35,72,73].

The blending ratio had the most significant effect on the board properties. The presence of lightweight non-woody particles, such as leaves and porous bark, lowers the overall density of the biomass, resulting in composites with lower densities as the biomass content increases. The lower biomass density results in a disproportionately higher biomass volume per weight, which reduces the potential of the plastic to wet, infiltrate, and encapsulate the proportionately larger volume of the biomass. Since the resistance of WPCs to moisture sorption, and an effective stress transfer is reliant on the interaction between the stiffer hydrophilic biomass and the ductile hydrophobic polymer, the pathways for moisture movement into the composites and stress concentration points are higher in an incomplete biomass particle enclosure. Consequently, the blending ratio between wood and plastic needs to be adjusted according to the intended application. At a 50% biomass ratio, there is enough plastic to provide moisture resistance, but the boards are less rigid and have a higher density, which is not desirable for insulation boards. Boards with a 60% biomass fraction display a good balance between moisture resistance, strength, and medium density, with thermo-acoustic insulation benefits for interior use [74].

## 5. Conclusions

WPC boards were made in a cost-effective way, without prior processing of the biomass, such as debarking, separating non-woody and woody parts, or the use of additives, such as compatibilisers. The recycled plastic was the lowest grade—and therefore cheapest—rLDPE. The resulting boards had physical and mechanical properties comparable to commercial products used for insulation purposes and met all the requirements for non-structural interior applications in buildings. The long-term WA and TS results suggest that the panels may be used in high-humidity interior environments, such as kitchens, without much risk of biodegradation and deformation, while the density and mechanical properties make them suitable for interior wall cladding and ceiling boards. All the investigated processing factors—blending ratio, particle size, press time, and temperature—were found to significantly affect the board properties, albeit to varying degrees. The blending ratio is the most important determinant of physical and mechanical properties. A higher press temperature and longer press times lower the viscosity of the plastic and improve the melt-flow rates, while also re-crystallising the cellulose fibres. This gives rise to stronger bonds between the plastic and biomass and a better encapsulation of the biomass particles. However, the maximum temperature is limited to prevent thermal degradation of the biomass. WPC boards made with smaller particle fractions showed better physical and mechanical properties due to a better encapsulation of the biomass particles by the plastic matrix, which leads to improved hydrophobic properties. However, the improvement in the mechanical properties was not as notable.

Ultimately, boards made with a 60% biomass ratio and pressed at 180 °C for 30 min showed the best balance between mechanical and physical properties for interior applications. The obtained density is sufficient but not too high to allow for a good thermo-acoustic insulation.

## Figures and Tables

**Table 1:** Blending ratios and time–temperature-mix treatments of WPCs at ***S*** (0.31 mm) and ***L*** (0.47) particle sizes.

Blending Ratio (wt. %)	Particle size *S*			Pressure (kg/cm^2^)	Particle Size *L*		
	Press Temperature (°C)	Press Time (min)	Sample Code		Press Temperature (°C)	Press Time (min)	Sample Code
50:50	150	10	5SA	2500	150	10	5LA
		30	5SB			30	5LB
	180	10	5SC		180	10	5LC
		30	5SD			30	5LD
60:40	150	10	6SA		150	10	6LA
		30	6SB			30	6LB
	180	10	6SC		180	10	6LC
		30	6SD			30	6LD
70:30	150	10	7SA		150	10	7LA
		30	7SB			30	7LB
	180	10	7SC		180	10	7LC
		30	7SD			30	7LD

**Table 2:** Compositional analysis of the wood of *A. saligna*, *A. mearnsii*, and *E. camaldulensis*.

Parameters (%)	Biomass Type		
	*A. saligna*	*A. mearnsii*	*E. camaldulensis*
Lignin	19.69 (0.48)	18.92 (0.57)	21.14 (1.96)
Hemicelluloses	15.18 (0.69)	16.24 (0.10)	17.36 (1.23)
Cellulose	41.97 (2.54)	35.55 (0.49)	28.74 (2.34)
Water Extractives	7.62 (0.41)	6.02 (1.22)	4.85 (0.78)
Ethanol Extractives	0.18 (0.19)	0.15 (0.09)	0.18 (0.04)
Total Extractives	7.80 (0.19)	6.17 (0.92)	5.03 (0.64)
Ash	1.07 (0.47)	1.30 (0.76)	0.93 (0.08)

Values in parenthesis are the standard deviations.

**Table 3:** Board density (ρ) and WA and TS of the WPCs made with small (*S*) and large (*L*) particle sizes at different blending ratios after 2 h, 24 h, and 672 h.

Treatment	Sample Code ^a^	ρ (g/cm^3^)	WA (h)			TS (h)			Sample Code ^a^	ρ (g/cm^3^)	WA (h)			TS (h)		
			2	24	672	2	24	672			2	24	672	2	24	672
**A**	5SA	1.61	3.27 (0.23)	14.43 (1.34)	30.76 (2.44)	4.39 (0.39)	7.03 (0.60)	9.60 (0.55)	5LA	1.49	3.68 (0.17)	11.71 (0.56)	29.64 (0.83)	2.56 (0.27)	4.30 (0.37)	8.40 (0.68)
	6SA	0.93	5.27 (0.12)	12.54 (0.38)	33.96 (0.62)	1.42 (0.06)	5.10 (0.20)	9.30 (0.30)	6LA	0.94	5.70 (0.27)	19.01 (1.44)	37.46 (1.86)	3.79 (0.25)	6.23 (0.52)	8.94 (0.58)
	7SA	0.96	10.69 (0.19)	22.81 (0.28)	37.05 (0.45)	5.70 (0.15)	8.74 (0.40)	11.46 (0.32)	7LA	0.76	11.40 (0.18)	22.63 (0.34)	37.40 (1.44)	4.76 (0.05)	8.27 (0.07)	12.06 (0.12)
**B**	5SB	1.52	2.84 (0.15)	10.10 (0.33)	30.53 (0.83)	3.99 (0.14)	4.54 (0.42)	6.91 (0.60)	5LB	1.54	2.50 (0.08)	10.37 (0.54)	28.18 (1.09)	0.71 (0.05)	2.61 (0.15)	6.26 (0.35)
	6SB	1.01	2.65 (0.06)	9.23 (0.20)	30.30 (0.59)	0.65 (0.03)	3.57 (0.16)	6.49 (0.31)	6LB	0.97	2.95 (0.08)	10.63 (0.28)	35.58 (0.85)	0.43 (0.02)	3.70 (0.20)	7.19 (0.32)
	7SB	0.93	8.72 (0.44)	22.65 (1.68)	34.09 (2.39)	3.89 (0.22)	7.17 (0.53)	9.61 (0.71)	7LB	0.88	8.30 (0.22)	21.07 (0.95)	34.87 (1.76)	2.53 (0.05)	6.87 (0.23)	10.79 (0.31)
**C**	5SC	1.46	2.79 (0.10)	6.83 (0.31)	27.39 (2.28)	3.15 (0.34)	4.57 (0.47)	6.86 (0.63)	5LC	1.39	2.97 (0.12)	8.62 (0.36)	27.36 (0.74)	0.65 (0.04)	1.95 (0.10)	5.90 (0.27)
	6SC	1.12	2.73 (0.05)	9.77 (0.11)	31.02 (0.25)	1.30 (0.06)	4.49 (0.17)	6.95 (0.26)	6LC	0.97	3.82 (0.09)	11.86 (0.23)	32.67 (0.86)	2.30 (0.16)	4.91 (0.35)	7.55 (0.47)
	7SC	0.97	5.24 (0.11)	14.79 (0.34)	29.76 (0.51)	3.51 (0.18)	6.97 (0.25)	9.65 (0.34)	7LC	0.89	8.91 (0.29)	21.72 (0.88)	35.87 (1.71)	4.12 (0.17)	7.95 (0.22)	11.46 (0.33)
**D**	5SD	1.41	1.32 (0.04)	5.20 (0.17)	19.85 (0.82)	0.58 (0.03)	1.87 (0.09)	6.39 (0.29)	5LD	1.47	2.43 (0.07)	8.53 (0.23)	24.45 (0.85)	0.50 (0.04)	1.70 (0.12)	5.64 (0.37)
	6SD	0.94	2.32 (0.07)	8.09 (0.28)	27.28 (0.81)	1.03 (0.04)	2.39 (0.09)	6.25 (0.16)	6LD	1.06	2.26 (0.04)	8.50 (0.17)	27.88 (0.62)	1.14 (0.05)	3.43 (0.15)	5.84 (0.21)
	7SD	0.99	4.89 (0.12)	14.24 (0.30)	27.96 (0.67)	2.43 (0.06)	4.51 (0.14)	9.04 (0.25)	7LD	0.89	6.62 (0.23)	19.48 (0.68)	30.18 (1.07)	2.39 (0.07)	6.21 (0.21)	10.23 (0.26)

**^a^** refer to Table 1 for the WPC treatment composition and corresponding codes. (Values in parenthesis are the standard deviations).

## Data Availability

All data is available upon request.
